# Genetic and Phenotypic Characterization of a *Salmonella Enteritidis* ST11 Clinical Isolate Carrying *bla*_NDM-13_ in Jiaxing City, China

**DOI:** 10.3390/antibiotics15040381

**Published:** 2026-04-09

**Authors:** Ping Li, Weiming Yang, Zhongwen Chen, Henghui Wang, Miaomiao Jia, Xuejuan Liu, Yong Yan, Guoying Zhu

**Affiliations:** 1 Jiaxing Center for Disease Control and Prevention, Jiaxing 314050, China; pljxcdc@163.com (P.L.);; 2 Pediatric Department, Affiliated Hospital of Jiaxing University, Jiaxing 314000, China

**Keywords:** *Salmonella Enteritidis*, carbapenems, *bla*_NDM-13_, genetic analysis, comparative analysis, mobile genetic elements

## Abstract

Background/Objectives: Multidrug-resistant *Salmonella* enterica serovar *Enteritidis*, especially those isolated from humans, remains a public concern. In the present study, *S*. *Enteritidis* strain 31404 was obtained clinically from a fecal sample of a fifteen-year-old girl, who was positive for *bla*_NDM-13_. Methods: Antibiotic susceptibility testing and whole genome sequencing were performed. Core genome MLST and hierarchical clustering (HierCC) were performed using EnteroBase. Population structure analysis of 57 *S*. *Enteritidis* isolates collected between 2023 and 2025 in Jiaxing city was conducted. A comparative structure analysis of *bla*_NDM-13_-positive plasmids was also performed. Results: *S*. *Enteritidis* strain 31404 was resistant to 13 antimicrobial agents. We found that strain 31404 belonged to ST11 and carried resistance genes, such as *bla*_NDM-13_, *bla*_CTX-M-14_, *ble*_MBL_, *fosA3*, *qnrS*, and *tet* (A). *bla*_NDM-13_ was located on an IncI1-I (α) plasmid designated as p31404-NDM13. *S*. *Enteritidis* isolate 31404 was closely related to PNUSAS514422, which was isolated from the United States in 2025. Comparative genetic environment related to *bla*_NDM-13_-positive plasmids available in the NCBI database indicates that ΔTn*125*-mediated contexts were commonly associated with *bla*_NDM-13_. IS*1294* (IS*91* family), which replaces IS*Aba125*, is likely to mobilize *bla*_NDM-13_. Conclusions: The findings in this study provide insights into the molecular characterization and diversification of *bla*_NDM-13_. The identification of *bla*_NDM-13_-containing transferable plasmids in different serotypes of *Salmonella* isolates (such as *S*. *Rissen*, *S*. *Typhimurium*, and *S. Enteritidis*) in different cities in China highlights the risk of the spread of carbapenem-resistant genes among *Salmonella* isolates.

## 1. Introduction

*Salmonella* infection is a major public health concern and contributes substantially to the global burden of foodborne disease. *Salmonella* causes about 95 million infections, resulting in approximately 150,000 deaths globally [1]. Over 2600 *Salmonella* serotypes have been identified. For Non-Typhoid *Salmonella* (NTS), *Salmonella Enteritidis* (*S*. *Enteritidis*) spreads in a pandemic-like manner [2] and together with *S*. enterica serovar *Typhimurium*, consistently ranks among the top two serotypes associated with human disease worldwide [3]. The clinical outcome of salmonellosis from *S*. *Enteritidis* infection typically causes gastrointestinal-related symptoms. Children, senior citizens, and people with impaired immunity are populations that are more vulnerable to unfavorable consequences [4].

Many virulence factors play diverse roles in the pathogenesis of *Salmonella* infections. *Salmonella* pathogenicity islands (SPIs) are genomic clusters in chromosomes that increase infection risks and lead to severe disease manifestations [5]. It has been estimated that *Salmonella* causes nearly 100,000 antimicrobial-resistant infections annually in the United States [6]. The serious adverse effects and consequences of antibiotic-resistant *Salmonella* infections are bloodstream infections, meningitis, septicemia, and high hospitalization and fatality rates [7,8]. The multidrug resistance (MDR) of *Salmonella* has raised significant concerns worldwide because of rising morbidity, mortality, and economic costs [9]. In the United States, an increased incidence of resistance to clinically important antimicrobials (ampicillin and ceftriaxone) or nonsusceptibility to ciprofloxacin has been identified among *S*. *Enteritidis* from 2015 to 2016, compared with the incidence from 2004 to 2008 [10]. The World Health Organization has declared that antibiotic-resistant *S*. *Enteritidis* is a critical-priority bacterium [11].

Plasmid-mediated resistance to third- and fourth-line antibiotics, including colistin (*mcr* genes) [12], β-lactam antibiotics, and azithromycin (*mph* or *erm* (B) genes) in *Salmonella* remained a public health issue. β-lactamases, the major resistance determinant for β-lactam antibiotics in Gram-negative bacteria, are divided into Ambler classes A, B, C, and D based on their protein sequence homology [13]. Class B β-lactamases, the metallo-β-lactamases (MBLs), can hydrolyze substrates through one or two essential zinc ions in the active-site groove. MBLs, including IMP-type [14], VIM-type [15], and NDM-type [16] enzymes, have been among the most prevalent carbapenemases among carbapenem-resistant Enterobacteriaceae around the world [17].

*bla*_NDM_, the class B β-lactamases, can hydrolyze all classes of β-lactam antibiotics (penicillins, cephalosporins, and carbapenems), except monobactams [18]. *bla*_NDM-1_ was initially isolated from a carbapenem-resistant *Klebsiella pneumoniae* from an Indian hospital in 2009 [16]. Since then, up to 90 distinct New Delhi metallo-β-lactamase (NDM) variants have been identified globally [19]. In *Salmonella*, the highest number of reports corresponds to NDM-1, which has been detected in a variety of serovars, such as *Senftenberg* [20], *Stanley* [21], *Agona* [22], and *Mbandaka* [23] from clinical isolates and *Indiana* [24] and *Corvallis* [25] from animals and foods. The monophasic variant of *S*. *Typhimurium* [26], *S*. *Idikan*, and *S*. *London* [27] proved to be positive for the *bla*_NDM-5_ gene. Additionally, an extensively drug-resistant *S*. *Indiana* strain harbored chromosomal *bla*_NDM-9_ [28]. NDM-13 has two amino acid substitutions (D95N and M154L) compared with NDM-1, resulting in the increased hydrolytic activity against cefotaxime [29]. NDM-13 was first identified in the chromosome of a multidrug-resistant *Escherichia coli* (*E. coli*) clinical isolate in Nepal. After that, *bla*_NDM-13_ was also found on the plasmids of clinically isolated Enterobacteriaceae. To date, NDM-13 has been identified in five *E*. *coli* strains obtained from Nepal (n = 1) [29], China (n = 3), and Korea (n = 1) [30], and one *S*. *Rissen* isolate [31], and one *S*. *Typhimurium* ST19 isolate [32].

β-lactamase-encoding genes can be acquired horizontally by different means, but mainly by plasmid acquisition. The *bla*_NDM-1_ gene in *Salmonella* previously appears on the IncA/C-, IncL/M-, IncX3-, and IncHI2-type plasmids. Different types, including IncFII, IncHI2, IncFIB, and IncX3, were also reported to be positive for *bla*_NDM-5_ [33,34]. *Salmonella* strains carrying *bla*_NDM-13_ are rare, but IncI1 plasmids carrying *bla*_NDM-13_ have still been discovered in *S*. *Rissen* [31] and *S*. *Typhimurium* ST19 [32].

Various studies demonstrated an increase in first-line agents, such as third-generation cephalosporins and fluoroquinolones, in *Salmonella* [35]. In 2017, the WHO considered d fluoroquinolones-resistant *Salmonella* spp. as pathogens for which novel antibiotics are urgently required [36]. In Enterobacterales, including *Salmonella*, quinolone resistance typically develops from the accumulation of chromosomal mutations in the quinolone resistance-determining region (QRDR) (*gyr*A, *gyr*B, *par*C, and *par*E genes) and plasmid-mediated quinolone resistance (PMQR) mechanisms. Three types of PMQR mechanisms have been identified: (1) quinolone resistance proteins (Qnr), (2) *aac (6′)-Ib-cr* resistance mechanism, and (3) plasmid-mediated resistance by OqxAb and QepA efflux systems [37,38,39]. The Qnr, including *qnr*A, *qnr*B, *qnr*C, *qnr*D, *qnr*E, *qnr*S, and *qnr*VC, was the major PMQR gene among NTS human isolates [40]. The *qnr* genes and extended-spectrum β-lactams (ESBLs) resistance gene usually coexist on the same plasmid, as also identified in some studies, which may pose a significant threat to antimicrobial therapy [41,42].

In the present study, an MDR *Salmonella* strain 31404 was found to be positive for *bla*_NDM-1_ and belonged to *S*. *Enteritidis*. Detection of carbapenemase resistance in *S*. *Enteritidis* is a cause of concern, as this serotype remains the most common S. enterica serovar in many parts of the world. Hence, we characterized the antibiotic resistance of strain 31404 using phenotypic susceptibility data. Whole-genome sequencing (WGS) was used to analyze the population structure of *S*. *Enteritidis* isolates collected in Jiaxing city, China. Comparative genetic analysis was also used to clarify the evolution of *bla*_NDM-13_-positive plasmids in different species.

## 2. Results

### 2.1. General Features of Strain 31404

Strain 31404 was identified as *S*. *Enteritidis* ST11 by multilocus sequence typing after WGS analysis. We found that this strain had MDR against 13 antimicrobial agents belonging to six distinct classes, excluding susceptibility to azithromycin, gentamicin, amikacin, tigecycline, trimethoprim-sulfamethoxazole, chloramphenicol, and florfenicol and intermediate resistance to imipenem, meropenem, polymyxin, and colistin (Table 1). In addition to *bla*_NDM-13_, strain 31404 carried genes that mediate resistance to β-lactams (*bla*_CTX-M-14_), bleomycin (*ble*_MBL_), fosfomycin (*fos*A3), ciprofloxacin (*qnr*S), and tetracycline [*tet* (A)]. Plasmid replicon types, such as IncFIB (S), IncFII (S), IncX1, IncI1-I (α), IncQ1, and pXuzhou21, were identified in strain 31404. Based on the annotation of the virulence factor database (VFDB), strain 31404 harbored virulence genes, including fimbrial adherence determinants, nonfimbrial adherence determinants (*mis*L), iron uptake (*ent*AB and *fep*G), macrophage inducible gene (*mig*-14), motility (e.g., *flg*CGHI), type III secretion systems (T3SS), and serum resistance (*omp*A) (Table 2).

### 2.2. The Population Structure of S. Enteritidis Strains

**Figure 1:**
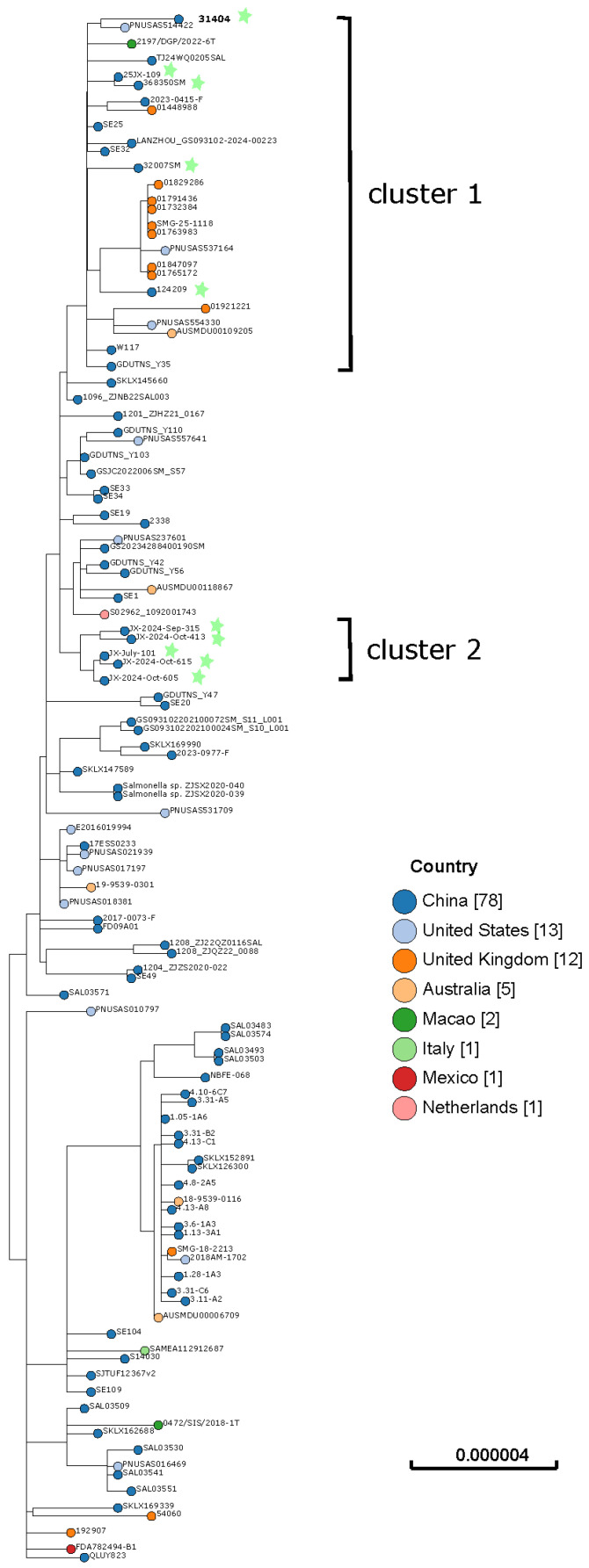
cgMLST analysis of 113 *S. Entertidis* HC20-7952 strains obtained in Enterobase in March 2026. The “n” in the brackets represents the number of isolated strains for each country. Blue circles with star symbols indicate isolates from this study.

In this study, *S. Enteritidis* isolate 31404 clustered within HC20-7952. In the Enterobase, we found 277 HC20-7952 sequences. After filtering, 133 sequences were finally selected for the cgMLST analysis (Table S1). As shown in Figure 1, 31404 was closely related to PNUSAS514422, which was isolated from the United States in 2025. Also, 25JX-109, 368350SM, 32007SM, and 124249 collected in this study were clustered into the same group with 31404 and belonged to different HC2. In this cluster, strains collected from China (n = 7), the United Kingdom (n = 9), the United States (n = 3), Australia (n = 1), and Macao (n = 1) were also identified. Notably, five strains collected from Jiaxing city in 2024 fell within a single clade, comprising isolates collected from two regions of Jiaxing in different months.

All 57 *S*. *Enteritidis* isolates collected in this study belonged to ST11. The single-nucleotide polymorphism (SNP) analysis showed clustering of several isolates from different years, suggesting phylogenetic relatedness. The SNPs of the 57 isolates ranged from 0 to 163. Strain 31404 exhibited a close genetic relationship with strain 25JX-109 (SNP = 17). Information regarding these strains is listed in Table 3.

At least one antibiotic-resistant gene was found in 42 of 57 *S*. *Enteritidis* isolates. Among them, *bla*_TEM-1B_ was the most prevalent, followed by *aph (3″)-Ib* and *sul2*. Other β-lactamase genes were identified, including *bla*_CTX-M-14_ in four isolates, and *bla*_CTX-M-55_ in two.

**Figure 2:**
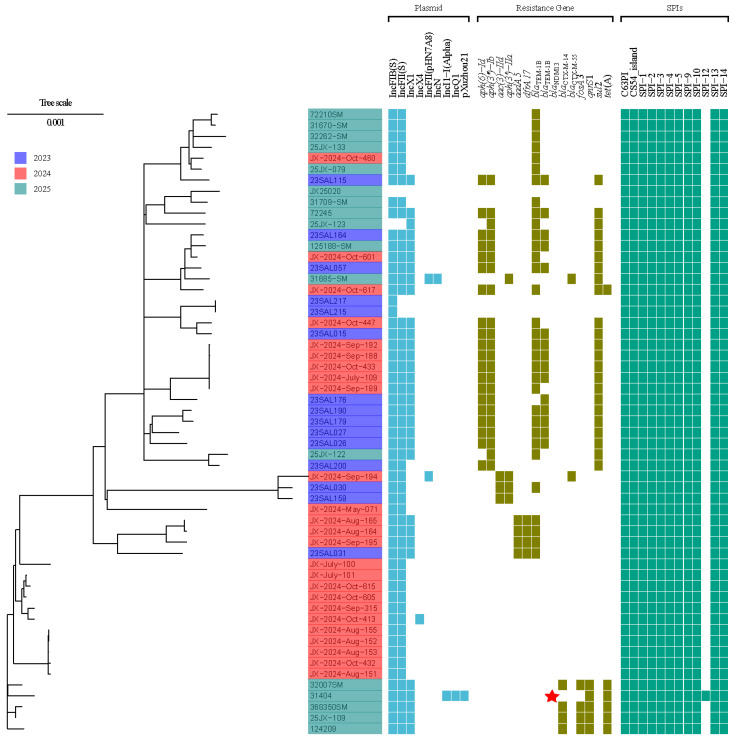
SNP analysis of 57 *S*. *Enteritidis* isolates from Jiaxing City, China. The presence of plasmid replicon sequence, resistance genes, and SPIs is indicated in different colors. The presence of *bla*_NDM13_ in 31404 was indicated with red stars.

In the studied isolates, two types of SPIs were detected. Most strains (96.7%, 56/57) were uniformly carrying C63PI, CS54_island, SPI-1, SPI-2, SPI-3, SPI-4, SPI-5, SPI-9, SPI-10, SPI-13, and SPI-14. Additionally, SPI12 was only found in strain 31404. IncFIB (S)-IncFII (S)-IncX1 (52.6%, 30/57) and IncFIB (S)-IncFII (S) (40.4%, 23/57) were the most common incompatibility group profiles among the 57 isolates (Figure 2).

### 2.3. Characteristics of bla_NDM-13_-Positive Plasmids

**Figure 3:**
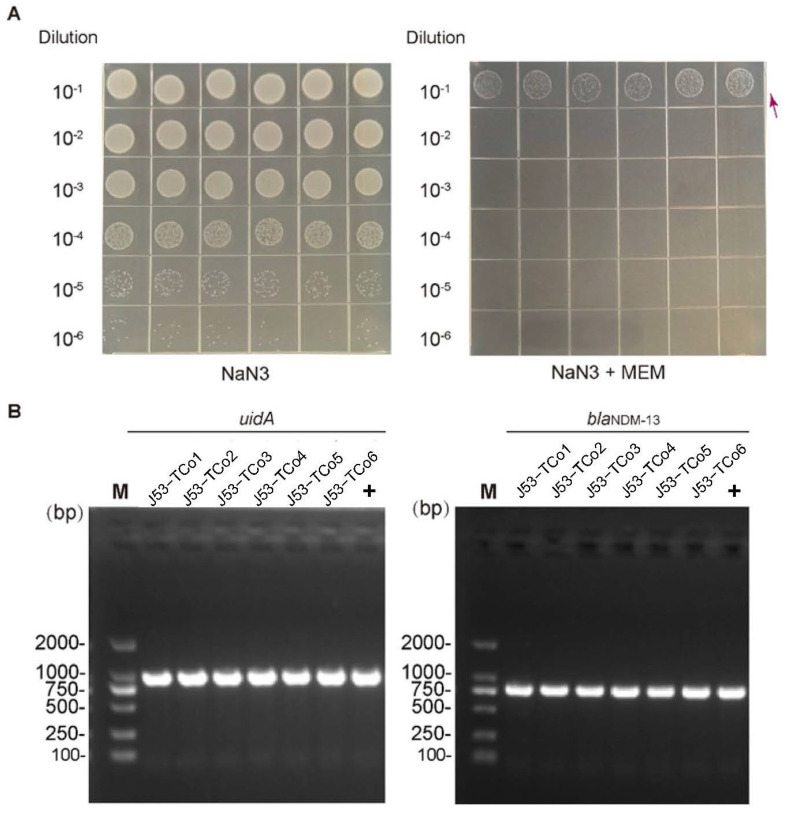
(**A**) Conjugation assay of isolate 31404 using sodium azide-resistant *E. coli* J53 as the recipient. From left to right: selection results on agar containing sodium azide alone, and a combination of sodium azide and meropenem. Arrows indicate transconjugant colonies. (**B**) PCR verification of transconjugants selected on sodium azide and meropenem. The *uidA* gene serves as an *E. coli*-specific marker. The + sign denotes the positive control.

WGS showed that blaNDM-13 and bleMBL were located on an IncI1-I (α) plasmid designated as p31404-NDM13, which is 88,385 bp in length with an average GC content of 50.4%. The mobility analysis based on plasmid sequence showed that p31404-NDM13 was predicted as conjugative. To investigate the horizontal transfer capacity of p31404-NDM13, conjugation assays were also performed. Results demonstrated that p31404-NDM13 was successfully transferred to the recipient strain *E. coli* strain J53 and conferred meropenem resistance (Figure 3).

**Figure 4:**
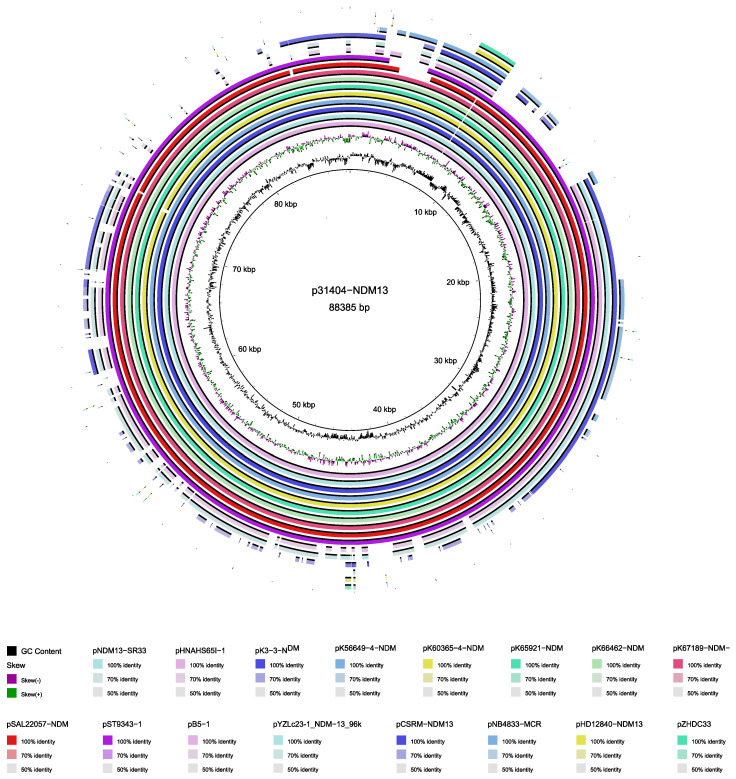
Sequence alignment between plasmid p31404-NDM13 and 16 other *bla*_NDM-13_-carrying plasmids retrieved from the Genebank database. The figure was generated by BRIG.

As of 28 December 2025, we investigated all 16 *bla*_NDM-13_-positive plasmid sequences publicly available in NCBI, as well as p31404-NDM13 in the present study, and observed that these samples were collected between 1984 and 2025 (Table 4). All strains of these plasmids originated in China, except for one strain of unknown origin. Most of these plasmids were identified from *E. coli* (41.2%, 7/17), followed by *K. pneumoniae* (35.3%, 6/17), and *Salmonella* spp. (23.5%, 4/17). The IncI1-I (α) plasmid remained a key mediator of *bla*_NDM-13_ (70.6%, 12/17). Sequence alignments demonstrated that p31404-NDM13 was identical to pNDM13-SR33, which was identified from a clinical *S*. enterica serovar *Rissen* strain in 2021. It also showed high similarity with *bla*_NDM-13_-carrying plasmids isolated from *K. pneumoniae* (100% coverage and 99.99% identity) (Figure 4). Resistance genes *bla*_NDM-13_ and *ble*_MBL_ were the two carried by the IncI1-I (α) plasmid in *K. pneumoniae*, *Salmonella* spp., and one *E. coli* strain. Several plasmids in *E. coli*, including IncB/O/K/Z, IncI1-I (α), IncFIB (AP001918)/IncFII (pHN7A8), and IncX3, were found to be positive for *bla*_NDM-13_. Other resistance genes, such as *bla*_SHV-12_ and *fosA3*, were also carried by *bla*_NDM-13_-carrying plasmids in *E. coli* (Table 4).

### 2.4. Genetic Context Comparison of the bla_NDM-13_ Region in bla_NDM-13_-Positive Plasmids

**Figure 5:**
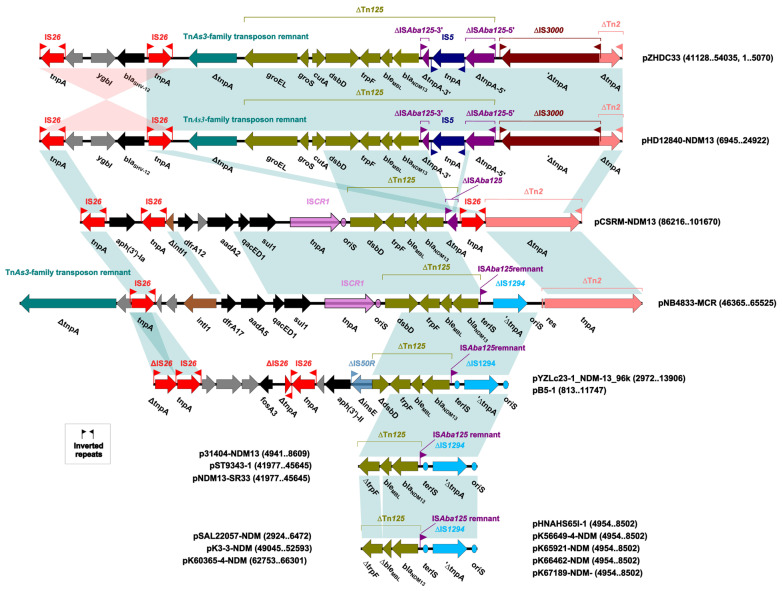
Genetic environments related to the *bla*_NDM-13_ gene in bacterial plasmids. The figure was generated by Easyfig. Confirmed and putative open reading frames (ORFs) are indicated by block arrows and their orientations with different colors.

The genetic environment related to *bla*_NDM-13_ in p31404-NDM13 is shown in Figure 5. Structural analysis revealed that the *bla*_NDM-13_ regions from 17 plasmids were classified into seven groups. The *bla*_NDM-13_ region in pHD12840-NDM13 and pZHDC33 was the longest and contained several truncated transposons (ΔTn*As3*-family transposon, ΔTn*125*, and ΔTn*2*) and insertion sequences (IS*26*, IS*5*, and ΔIS*3000*). The *bla*_NDM-13_ gene was carried by a truncated Tn*125* composed of ∆IS*Aba125*-5′—IS*5*—∆IS*Aba125*-3′—*bla*_NDM-13_—*ble*_MBL_—*trpF*—*dsbD*—*cutA*—*gros*—*groEL*. Compared with pHD12840-NDM13/pZHDC33, the IS*Aba125* upstream of *bla*_NDM-13_ in pCSRM-NDM13 was interrupted by IS*26*. An integron carrying *dfrA12*, *aadA2* (pseudogene), *qacED1*, and *sul1*, as well as IS*CR1*, was located downstream of *dsbD*. In pNB4833-MCR, ΔIS*1294*, rather than IS*26*, was present upstream of *bla*_NDM-13_. Also, an integron carrying *dfrA17*, *aadA5*, *qacED1*, and *sul1*, as well as the insertion sequence IS*CR1*, was located downstream of *dsbD*. The *bla*_NDM-13_ region in pYZLc23-1_NDM-13_96k/pB5-1, was carried by a ΔTn*125* (∆IS*Aba125*—*bla*_NDM-13_—*ble*_MBL_—*trpF*—∆*dsbD*) flanked by ΔIS*1294* and ΔIS*50R*. The corresponding region in p31404-NDM13/pST9343-1 was arranged by ΔIS*1294*—∆IS*Aba125*—*bla*_NDM-13_—*ble*_MBL_—∆*trpF*. The relative region in pSAL22057-NDM/pHNAHS65I-1/pK3-3-NDM was similar to that in p31404-NDM13, with a truncated *ble*_MBL_ between *bla*_NDM-13_ and ∆*trpF*.

## 3. Discussion

*Salmonella* enterica is a major cause of bacterial gastroenteritis worldwide. Infections caused by *Salmonella* are commonly observed as acute gastroenteritis [43]. *S*. *Enteritidis* ranked as the first serotype of all foodborne *Salmonella* isolates collected from the laboratories of 37 countries between 2001 and 2007 [44]. The emergence and spread of antimicrobial resistance (AMR) in *Salmonella* have posed a serious public health challenge. Here, we report the identification of an NDM-13-positive *Salmonella* strain 31404 clinically isolated from a child in Jiaxing, China. The formation of the *bla*_NDM-13_ region results from diverse recombination events involving multiple mobile genetic elements (MGEs). We unveiled the genome structure of *S*. *Enteritidis* isolates in Jiaxing. The data generated here and made publicly available provide a basis for further work on the evolution and transmission of *bla*_NDM-13_ across diverse *Salmonella* serotypes.

*S*. *Enteritidis* sequence type (ST)11 is the most common sequence type, geographically widespread [45,46]. ST183, ST1925, ST1974, and ST5895 have been found in this serotype [47]. In the present study, all 57 *S*. *Enteritidis* strains were identified as ST11 across various sectors (human, food, and environment), which may result from the scale and scope of the study. Bacterial virulence-related genes present in *Salmonella* efficiently initiate and accelerate the development of foodborne illnesses. The *invA* gene within SPI-1 is a critical factor in host cell penetration. The *spiC* encoding SPI-2 produces T3SS-2 translocated effectors [48]. SPI-12 was only identified in strain 31404, whereas it is absent in the other 56 *S*. *Enteritidis* strains. SPI-12 contributes to the fitness of *S*. *Typhimurium* in vivo and encodes a remnant phage known to contribute to bacterial virulence and improve fitness in the host [49]. The clustering of isolates from different sources and location consistent with the result that *S*. *Enteritidis* was spread in a pandemic-like manner [2].

Several β-lactamase genes, such as those for extended-spectrum β-lactamase (ESBL) and AmpC-like lactamases, including *bla*_CTX-M-55_, *bla*_TEM-1B_, and *bla*_CMY-2_, were abundant in the China subclade as well as in a clade from sub-Saharan Africa [47]. Notably, these genes were also reported as the most common ESBL genes carried in the ceftriaxone-resistant isolates in China from 2007 to 2016 [50]. *bla*_TEM-1B_ followed by *bla*_CTX-M-14_ were the most frequently detected β-lactamase genes among 57 *S*. *Enteritidis* strains from Jiaxing, consistent with previous studies. The first report of *bla*_NDM-13_ in *Salmonella* was from a clinical MDR strain *S*. *Rissen* [31]. To our knowledge, the present study is the first report of *bla*_NDM-13_ in *S*. *Enteritidis*.

*Salmonella* spp. are important pathogens because they are very adaptive to antimicrobial selection pressure [51]. Plasmids play a role in the transmission of AMR in *Salmonella* spp. [52]. Plasmid replicons IncFII (S) and IncFIB (S) were the most frequently detected in *S*. *Enteritidis* isolates in Jiaxing, consistent with findings from a study conducted by Ewelina Kamińska in Poland [47,53]. As found in the present study, most strains carried two to three replicons. The coexistence and horizontal dissemination of plasmids in *Salmonella* spp. provide significant evolutionary advantages by expanding the host range and enhancing bacterial persistence across diverse ecological niches. For strain 31404, the IncI1-I (α) replicon was associated with the *bla*_NDM-13_. These plasmids have previously been identified as carrying *bla*_NDM-13_ in other *Salmonella* serovars, for example, *Rissen* [31] and *Typhimurium* [32], as well as in other species, such as *K. pneumoniae* and *E. coli*. Plasmid mobility analysis and conjugation experiments provide evidence that the IncI1 plasmid in this study acts as an efficient vector facilitating the acquisition of the *bla*_NDM-13_ gene in 31404.

Exceptionally dense and diverse MGEs, including transposons (Tn*3*, Tn*125* [54], and IS*26*-flanked pseudo composite transposons [55]), integrons (intI1), and ISs (IS*Aba125*, IS*3000*, IS*26* [56], IS*5*, and IS*CR1*) are thought to play important roles in *bla*_NDM_ dissemination through a multilayer process involving genetic recombination, transposition, conjugation, and transformation of plasmids [19,57]. The carbapenem resistance of strain 31404 was attributed to the presence of a plasmid-mediated β-lactamase gene, *bla*_NDM-13_, located on a truncated Tn*125* of a different length. The structure of Tn*125* was composed of IS*Aba125*—*bla*_NDM-1_—*ble*_MBL_—*trpF*—*dsbD*—*cutA*—*groES*—*gros*—IS*CR21*—IS*Aba125*, which was originally obtained from *Acinetobacter lwoffii* [56]. The Tn*125* transposon appears to have played an important role in early plasmid-mediated jumps of *bla*_NDM_. *bla*_NDM_ can be found in a variety of genomic contexts, and ΔTn*125*-mediated contexts were commonly associated with *bla*_NDM-13_ in the present study. Notably, IS*1294* (IS*91* family), which replaces IS*Aba125*, is likely to mobilize *bla*_NDM-13_ [58].

## 4. Conclusions

In conclusion, we reported the genetic and phenotypic characterization of a *Salmonella Enteritidis* ST11 clinical isolate carrying *bla*_NDM-13_. ΔTn*125*-mediated contexts were commonly associated with *bla*_NDM-13_ in the present study. Notably, IS*1294* (IS*91* family), which replaces IS*Aba125*, is likely to mobilize *bla*_NDM-13_. The findings in this study provide insights into the molecular characterization and diversification of *bla*_NDM-13_. Strain 31404 shows a high degree of similarity to a strain isolated from the United States, which indicates that we need to strengthen the monitoring of the spread of antibiotic resistance in *S. Enteritidis*. Additionally, the identification of *bla*_NDM-13_-containing plasmids in different serotypes of *Salmonella* isolates (such as *S*. *Rissen*, *S*. *Typhimurium*, and *S. Enteritidis*) from unrelated regions further validates the widespread dissemination of *bla*_NDM-13_ and carbapenem resistance in China.

## 5. Materials and Methods

### 5.1. Isolates Collection

Strain 31404 was isolated from a fecal sample of a fifteen-year-old girl. This patient was hospitalized due to occasional fever and diarrhea. Fecal samples from the patient were collected to isolate *Salmonella* spp. Within 4 h of collection, undiluted samples were streaked onto Columbia Blood Agar plates (CHROMagar, Shanghai, China) and cultured overnight at 37 °C. Suspected *Salmonella* spp. colonies were analyzed using matrix-assisted laser desorption/ionization–time of flight mass spectrometry. Serotyping was conducted using the slide agglutination method to detect somatic (O) antigen and flagellar (H) antigens (phases 1 and 2) following the White–Kaufmann–Le Minor Scheme. *Salmonella* Serotyping by Whole Genome Sequencing was confirmed using the Sequence query tool implemented in SeqSero 1.2 (https://genomicepidemiology.org/services/, accessed on 30 June 2025).

### 5.2. Antimicrobial Susceptibility Testing

Antimicrobial susceptibility testing (AST) of the following antimicrobial agents was performed to determine the minimum inhibitory concentration (MIC) of each using the microdilution method: ampicillin, ampicillin/sulbactam, amoxicillin/clavulanic acid, cefuroxime, ceftiofur, cefazolin, cefoxitin, cefotaxime, ceftazidime, cefepime, meropenem, imipenem, ertapenem, tetracycline, gentamicin, amikacin, trimethoprim/sulfamethoxazole, florfenicol, chloramphenicol, ciprofloxacin, nalidixic acid, colistin, polymixin, streptomycin, tigecycline, and azithromycin. The resistance breakpoints of ampicillin, ceftiofur, imipenem, meropenem, ertapenem, azithromycin, tetracycline, ciprofloxacin, trimethoprim/sulfamethoxazole, nalidixic acid, and chloramphenicol were determined in accordance with the principles outlined in relevant documents from the Clinical and Laboratory Standards Institute (CLSI) (M100-S32, M45-A3). Ampicillin/sulbactam, cefazolin, cefepime, cefotaxime, cefoxitin, ceftazidime, cefuroxime, gentamicin, and amikacin were determined in accordance with the European Committee on Antimicrobial Susceptibility Testing (EUCAST). Tigecycline was determined in accordance with the Food and Drug Administration (FDA). Colistin, polymixin, and florfenicol were interpreted in accordance with the “National Food Contamination and Hazardous Factor Risk Monitoring Work Manual 2025” (China National Center for Food Safety Risk Assessment, 2025). *Escherichia coli* ATCC 25922, *Enterococcus faecalis* ATCC 29212, and *Staphylococcus aureus* ATCC 29213 were used as quality control strains for AST.

### 5.3. Whole-Genome Sequencing Bioinformatics Analysis

Total genomic DNA was extracted from overnight (16–18 h) cultures of strains using the QIAamp DNA Mini Kit (Qiagen, Hilden, Germany) following the manufacturer’s instructions. WGS was performed using NextSeq 550 (Illumina, San Diego, CA, USA) platforms. The plasmid sequence of 31404 was performed using both the long-read Nanopore MinION (Nanopore, Oxford, UK) and the short-read NextSeq 550 (Illumina, San Diego, CA, USA) platforms. Briefly, for short-read sequencing, DNA libraries were prepared using the metagenomic DNA library construction kit (MD001T-P1, Hangzhou, China), purified using the magnetic bead purification kit (MATRIDX MD012, Hangzhou, China), and quantified using the KAPA Library Quantification Kit (07960140001, Roche, Switzerland). Following the manufacturer’s guidelines, the libraries were normalized, denatured, and diluted to a final concentration of 1.7 pM, then loaded onto the Illumina NextSeq 550 reagent cartridge (Mid Output Reagent Cartridge v2 300 cycles) for sequencing. For long-read sequencing, a sequencing library was constructed in accordance with the manufacturer’s protocols provided with the Sequencing Ligation Kit (Oxford Nanopore Technologies, Oxford, UK) and the library building kit (Baiyi Technology Co., Ltd., Hangzhou, China). Following quantitative dilution, the library was loaded onto the flow cell R10.4.1 (Oxford Nanopore Technologies, Oxford, UK) and sequenced using the P2 Solo protocol (Oxford Nanopore Technologies, Oxford, UK). The derived short reads and long reads were assembled using SPAdes (version 3.9.0) software. The average sequencing depth was >150. The completeness and contamination rate of the genome were evaluated using CheckM2 (V1.0.2) software (generally, a completeness of ≥ 90% and a contamination rate of ≤5% are required). With the *Salmonella* reference genome from the RefSeq database, the average nucleotide identity was calculated using Skani (V0.3.0) software (generally, an identity of ≥95% is required). The assembled sequence was annotated by RAST (Rapid Annotation using Subsystem Technology, https://rast.nmpdr.org/). The sequence type (ST) was determined using MLST (https://pubmlst.org/organisms?title=salmonella, accessed on 30 June 2025). Additionally, PlasmidFinder v2.1, ResFinder v4.7.2, and SPIFinder available at the Center for Genomic Epidemiology (https://genomicepidemiology.org/services/, accessed on 30 June 2025) were utilized for plasmid replicon, AMR genotypes, and SPI identification, respectively. The virulence genes were identified based on the annotation of the virulence factor database.

### 5.4. Genetic Analyses

*S*. *Enteritidis* 31404 was uploaded to the EnteroBase platform, where core genome multilocus sequence typing (cgMLST) and hierarchical clustering (HierCC) analyses were performed using built-in algorithms. Based on cgMLST allelic profiles, HierCC grouped the isolates at various hierarchical levels according to allele differences, such as HC2 and HC20. Each HC level represents the maximum number of allele differences allowed within a cluster, enabling the formation of genetically related groups. To identify genetically related isolates of the *S*. *Enteritidis* 31404 isolated in this study, the “Search Strains” function in EnteroBase was used to retrieve all genomes assigned to the same H20 clusters as of March 2026. Metadata of genomes belonging to the same HC2 clusters were filtered to remove redundant isolates. Briefly, isolates with incomplete metadata (e.g., missing country of origin or collection year) were excluded. Within each HC2 cluster, strains from the same country and the same year, depending on their source, will be retained with one sample. This strategy ensured the selection of representative and diverse isolates within each HC2.

Also, to comprehend the population structure of *S*. *Enteritidis* prevalent in Jiaxing City, the genome sequences of fifty-seven clinical and foodborne isolates collected between 2023 and 2025 were included for phylogenetic analysis. Data analysis was conducted using the BAIYI MicroGeno Platform (v5.4, Hangzhou Baiyi Technology Co., Ltd., http://www.baiyi-tech.cn/) for data analysis. SNPs were called using Snippy with default settings, except that only positions with base quality ≥Q30 and coverage ≥10× were retained, and SNPs in repetitive or low-complexity regions were excluded. Potential recombinant regions were identified and removed using Gubbins v3.3.5 (https://github.com/nickjcroucher/gubbins/, accessed on 29 December 2025) to ensure accurate phylogenetic inference. Maximum-likelihood (ML) phylogenetic trees were constructed using iqtree (v2.0.3 https://github.com/iqtree/iqtree2, accessed on 29 December 2025). The best-fit substitution model was selected with ModelFinder Plus (MFP), and branch support was evaluated using ultrafast bootstrap with 1000 replicates.

### 5.5. Plasmid Analysis

To further analyze the genetic environment and perform plasmid structural comparative studies, Easyfig v2.2.2 and BLAST Ring Image Generator (BRIG, v0.95) were used, respectively. Transposon and insertion sequence (IS) elements were scanned using the ISFinder database. BLASTn was used to determine the identity of the genetic environment between NDM-13-positive sequences.

### 5.6. Plasmid Mobility Prediction and Conjugation Experiment

The plasmid prediction was performed using MOB-suite, with identity and coverage thresholds set at 85% and 60%, respectively [59], which characterized the associated relaxase types, mating pair formation (MPF) systems, and origins of transfer (oriT), potentially involved in plasmid-mediated transfer.

The transferability of plasmids was evaluated via a filter membrane conjugation assay [60,61]. Briefly, the sodium azide-resistant *E. coli* strain J53 served as the recipient, while isolate 31404 was used as the donor. Donor and recipient cells were mixed at a 1:1 ratio. Transconjugants were selected on plates supplemented with sodium azide (100 mg/L) and meropenem (1 mg/L), then further verified by PCR targeting the *bla*_NDM_ and *uidA* genes. The specific primers used were NDM813-F (5′-ATGGAATTGCCCAATATTATGCA-3′), NDM813-R (5′-TCAGCGCAGCTTGTCGGC-3′), *uidA*-F (5′-CGAACTGAACTGGCAGACTATCC-3′), and *uidA*-R (5′-TAATGTTCTGCGACGCTCACA-3′).

### 5.7. Genbank Accession

The genomes of the Salmonella isolate 31404, 25 *S. Enteritidis* isolates from 2024, and 16 other *S. Enteritidis* isolates from 2025 reported in this study have been deposited in the National Center for Biotechnology Information and registered as BioProject numbers PRJNA1392682, PRJNA1439886, PRJNA1440394, and PRJNA1440466, respectively. Fifteen *S. Enteritidis* isolates from 2023 have been deposited in the National Center for Biotechnology Information previously [62]. The sequence of plasmid p31404-NDM13 was submitted to the GenBank database and assigned accession number PX754648.

## Figures and Tables

**Table 1:** Antimicrobial susceptibility of *S. Enteritidis* 31404 to a panel of antimicrobial agents.

Antimicrobial Class	Antimicrobial Agent (Abbreviation)	Breakpoint Interpretive Criteria (μg/mL)			MIC (μg/mL)	R/I/S
		S	I	R		
β-Lactam combination agents	Ampicillin/sulbactam (AMS)	≤8/4	16/8	≥32/16	>64	R
Penicillins	Ampicillin (AMP)	≤8	16	≥32	>64	R
Cephalosporins	Cefotaxime (CTX)	≤1	2	≥4	>8	R
	Ceftazidime (CAZ)	≤4	8	≥16	>32	R
	Cefoxitin (CFX)	≤8	16	≥32	>64	R
	Cefazolin (CFZ)	≤2	4	≥8	>32	R
	Ceftiofur (CEF)	≤2	4	≥8	>16	R
	Cefepime (CPM)	≤2	–	≥16	32	R
	Cefuroxime (CXM)	≤4	8	≥16	>64	R
Carbapenems	Imipenem (IPM)	≤1	2	≥4	2	I
	Meropenem (MEM)	≤1	2	≥4	2	I
	Ertapenem (ETP)	0.5	1	≥2	8	R
Macrolides	Azithromycin (AZM)	≤16	–	≥32	4	S
Aminoglycosides	Gentamicin (GEN)	≤4	8	≥16	≤1	S
	Amikacin (AMK)	≤16	32	≥64	≤4	S
	Streptomycin (STR)	–	–	–	≤4	NA
Tetracyclines	Tetracycline (TET)	≤4	8	≥16	>32	R
	Tigecycline (TIG)	≤2	4	≥8	≤0.25	S
(Fluoro) Quinolones	Nalidixic Acid (NAL)	≤16	–	≥32	>64	R
	Ciprofloxacin (CIP)	≤0.06	0.12∼0.5	≥1	2	R
Folate pathway inhibitors	Trimethoprim-sulfamethoxazole (SXT)	≤2/38	–	≥4/76	≤0.25	S
Phenicols	Chloramphenicol (CHL)	≤8	16	≥32	4	S
	Florfenicol (FFC)	≤4	8	≥16	4	S
Polymyxins	Polymixin (POL)	–	≤2	≥4	1	I
	Colistin (COL)	–	≤2	≥4	1	I

Note: R, resistant; I, intermediate; S, susceptible; NA, not applicable.

**Table 2:** Virulence-associated genes in 31404.

VF Classes	Virulence Factors	Genes
Fimbrial adherence determinants	Agf (thin aggregative fimbriae/curli)	*csg*ABCDEFG, *ste*ABCD
	Lpf (long polar fimbriae)	*lpf*ABCDE
	Type 1 fimbriae	*fim*ACDFHIWYZ
Non-fimbrial adherence determinants	MisL	*mis*L
	SinH	*sin*H
Iron uptake	Enterobactin	*ent*AB, *fep*G
Macrophage inducible gene	Mig-14	*mig-*14
Motility	Flagella	*che*BRWYZ, *flg*CGHI, *fli*GIMNQ, *flh*AC, *mot*A,
Secretion system	T3SS (SPI-1 encoded)	*inv*ABCEFGHIJ, *org*ABC, *prg*HIJK, *sic*AP, *sip*D, *spa*OPQRS, *hil*ACD, *iac*P, *iag*B,
	T3SS-1 translocated effectors	*avr*A, *sip*ABC/*ssp*ABC, *sop*ABDD2E2, *spt*P, *slr*P
	T3SS (SPI-2 encoded)	*ssa*CDEGHIJKLMNOPQRSTUVX, *ssc*AB, *sse*ABCDE, *ssr*AB,
	T3SS-2 translocated effectors	*pip*BB2, *sif*AB, *sse*FGIJK1L, *spi*C/*ssa*B
Serum resistance	OmpA (Outer membrane protein A)	*omp*A

**Table 3:** Information for 57 S. Enteritidis isolates collected in Jiaxing City, China.

Isolates	Jiaxing	Sample Type	Isolation_Source	Gender	Age	Collection Date	Serotype
23SAL176	Jiaxing	Clinical samples	feces	Male	51-00-00	14 June 2023	*S. Enteritidis*
23SAL057	Jiaxing	Clinical samples	feces	Female	55-00-00	20 June 2023	*S. Enteritidis*
23SAL026	Jiaxing	Clinical samples	feces	Female	54-00-00	22 April 2023	*S. Enteritidis*
23SAL027	Jiaxing	Clinical samples	feces	Male	62-00-00	6 May 2023	*S. Enteritidis*
23SAL015	Jiaxing	Clinical samples	feces	Male	39-00-00	7 May 2023	*S. Enteritidis*
23SAL031	Jiaxing	Clinical samples	feces	Male	07-00-00	8 April 2023	*S. Enteritidis*
23SAL030	Jiaxing	Clinical samples	feces	Female	41-00-00	4 May 2023	*S. Enteritidis*
23SAL115	Jiaxing	Clinical samples	feces	Female	17-00-00	10 September 2023	*S. Enteritidis*
23SAL158	Jiaxing	Food	Meat and meat product	/	/	17 October 2023	*S. Enteritidis*
23SAL179	Jiaxing	Clinical samples	feces	Female	58-00-00	10 July 2023	*S. Enteritidis*
23SAL190	Jiaxing	Clinical samples	feces	Female	02-09-00	15 October 2023	*S. Enteritidis*
23SAL200	Jiaxing	Clinical samples	feces	Male	80-00-00	5 October 2023	*S. Enteritidis*
23SAL215	Jiaxing	Clinical samples	feces	Male	00-09-29	1 October 2023	*S. Enteritidis*
23SAL217	Jiaxing	Clinical samples	feces	Male	00-10-01	9 October 2023	*S. Enteritidis*
23SAL164	Jiaxing	Clinical samples	feces	Female	38-00-00	12 August 2023	*S. Enteritidis*
JX-2024-Sep-194	Jiaxing	Clinical samples	feces	Male	2-08-00	6 July 2024	*S. Enteritidis*
JX-May-071	Jiaxing	Clinical samples	feces	Male	40-00-00	21 April 2024	*S. Enteritidis*
JX-2024-Oct-447	Jiaxing	Clinical samples	feces	Male	02-05-00	16 August 2024	*S. Enteritidis*
JX-2024-Oct-460	Jiaxing	Clinical samples	feces	Male	02-01-00	4 September 2024	*S. Enteritidis*
JX-2024-Oct-617	Jiaxing	Food	Meat and meat product	/	/	16 October 2024	*S. Enteritidis*
JX-2024-Oct-601	Jiaxing	Clinical samples	feces	Male	41-00-00	24 September 2024	*S. Enteritidis*
JX-2024-Sep-188	Jiaxing	Clinical samples	feces	Female	4-00-00	26 June 2024	*S. Enteritidis*
JX-2024-Oct-433	Jiaxing	Clinical samples	feces	Male	02-03-00	1 August 2024	*S. Enteritidis*
JX-2024-Sep-192	Jiaxing	Clinical samples	feces	Female	04-00-00	15 July 2024	*S. Enteritidis*
JX-2024-July-109	Jiaxing	Clinical samples	feces	Female	61-00-00	3 July 2024	*S. Enteritidis*
JX-2024-Sep-189	Jiaxing	Clinical samples	feces	Female	24-00-00	5 July 2024	*S. Enteritidis*
JX-2024-Sep-195	Jiaxing	Clinical samples	feces	Female	41-00-00	22 July 2024	*S. Enteritidis*
JX-2024-Aug-165	Jiaxing	Clinical samples	feces	Female	5-00-00	10 May 2024	*S. Enteritidis*
JX-2024-Aug-164	Jiaxing	Clinical samples	feces	Male	4-00-00	6 May 2024	*S. Enteritidis*
JX-2024-Oct-432	Jiaxing	Clinical samples	feces	Female	7-00-00	25 July 2024	*S. Enteritidis*
JX-2024-Aug-155	Jiaxing	Environmental samples	swabs from the inner wall of the refrigerator	/	/	29 July 2024	*S. Enteritidis*
JX-2024-Aug-153	Jiaxing	Others	Rags	/	/	29 July 2024	*S. Enteritidis*
JX-2024-Aug-152	Jiaxing	Clinical samples	feces	Female	6-00-00	28 July 2024	*S. Enteritidis*
JX-2024-Aug-151	Jiaxing	Clinical samples	feces	Female	7-00-00	28 July 2024	*S. Enteritidis*
JX-July-100	Jiaxing	Clinical samples	feces	Male	56-00-00	10 May 2024	*S. Enteritidis*
JX-2024-Oct-615	Jiaxing	Clinical samples	feces	Female	43-00-00	8 October 2024	*S. Enteritidis*
JX-2024-Oct-605	Jiaxing	Clinical samples	feces	Male	64-00-00	23 September 2024	*S. Enteritidis*
JX-2024-Sep-315	Jiaxing	Clinical samples	feces	Male	25-00-00	10 August 2024	*S. Enteritidis*
JX-2024-Oct-413	Jiaxing	Clinical samples	feces	Male	40-00-00	6 September 2024	*S. Enteritidis*
JX-July-101	Jiaxing	Clinical samples	feces	Male	78-00-00	25 May 2024	*S. Enteritidis*
124209	Jiaxing	Clinical samples	feces	Female	81-00-00	13 July 2025	*S. Enteritidis*
72210SM	Jiaxing	Clinical samples	feces	Male	68-00-00	2 September 2025	*S. Enteritidis*
72245	Jiaxing	Clinical samples	feces	Male	18-00-00	1 October 2025	*S. Enteritidis*
25JX-133	Jiaxing	Clinical samples	feces	Female	66-00-00	13 June 2025	*S. Enteritidis*
25JX-079	Jiaxing	Clinical samples	feces	Male	18-00-00	20 May 2025	*S. Enteritidis*
31404	Jiaxing	Clinical samples	feces	Female	15-00-00	13 June 2025	*S. Enteritidis*
32262-SM	Jiaxing	Clinical samples	feces	Male	1-00-00	29 September 2025	*S. Enteritidis*
368350SM	Jiaxing	Clinical samples	feces	Female	49-00-00	13 September 2025	*S. Enteritidis*
125188-SM	Jiaxing	Clinical samples	feces	Male	21-00-00	22 August 2025	*S. Enteritidis*
32007SM	Jiaxing	Clinical samples	feces	Male	3-00-00	16 January 2025	*S. Enteritidis*
JX25020	Jiaxing	Clinical samples	feces	Male	35-00-00	17 March 2025	*S. Enteritidis*
25JX-122	Jiaxing	Clinical samples	feces	Male	54-00-00	15 June 2025	*S. Enteritidis*
25JX-123	Jiaxing	Clinical samples	feces	Male	71-00-00	5 June 2025	*S. Enteritidis*
31709-SM	Jiaxing	Clinical samples	feces	Female	36-00-00	25 October 2025	*S. Enteritidis*
31670-SM	Jiaxing	Clinical samples	feces	Male	30-00-00	30 September 2025	*S. Enteritidis*
31685-SM	Jiaxing	Clinical samples	feces	Female	37-00-00	14 October 2025	*S. Enteritidis*
25JX-109	Jiaxing	Clinical samples	feces	Female	02-06-00	12 May 2025	*S. Enteritidis*

**Table 4:** Information on *bla*_NDM13_-carrying plasmids (last accessed 28 December 2025).

Plasmid Name	Plasmid Type	Serotype	Geo_Loc_Name	Collection Date	Isolation_Source	Compare with p31404-NDM13	Resistant Genes	Genebank Accession Number
p31404-NDM13	IncI1-I (Alpha)	*S. Enteritidis*	China: Jiaxing	13 June 2025	Feces specimen	/	*bla*_NDM13_	PX754648
pHNAHS65I-1	IncI1-I (Alpha)	*E. coli*	China	NA	Chicken meat	100%/100%	*bla*_NDM13_	MN219406
pNDM13-SR33	IncI1-I (Alpha)	*S. Rissen*	China: Xiamen, Fujian	14 September 2021	Stool	100%/100%	*bla*_NDM13_	CP092912
pK3-3-NDM	IncI1-I (Alpha)	*K. pneumoniae*	China: Chengdu	15 July 2024	Urine	100%/99.99%	*bla*_NDM13_	CP196483
pK56649-4-NDM	IncI1-I (Alpha)	*K. pneumoniae*	China: Beijing	March 2023	Blood	100%/99.99%	*bla*_NDM13_	CP168404
pK60365-4-NDM	IncI1-I (Alpha)	*K. pneumoniae*	China: Beijing	July 2023	Blood	100%/99.99%	*bla*_NDM13_	CP168412
pK65921-NDM	IncI1-I (Alpha)	*K. pneumoniae*	China: Beijing	2023	Sputum	100%/99.99%	*bla*_NDM13_	CP169925
pK66462-NDM	IncI1-I (Alpha)	*K. pneumoniae*	China: Beijing	2023	Blood	100%/99.99%	*bla*_NDM13_	CP169928
pK67189-NDM	IncI1-I (Alpha)	*K. pneumoniae*	China: Beijing	2023	Urine	100%/99.99%	*bla*_NDM13_	CP169934
pSAL22057-NDM	IncI1-I (Alpha)	*S. Stanly*	China: Zhengzhou	June 2022	Feces	97%/100%	*bla*_NDM13_	CP195667
pST9343-1	IncI1-I (Alpha)	*4,5,12:i:-*	China: Zhuhai	27 November 2023	Feces specimen	97%/100%	*bla*_NDM13_	CP162126
pB5-1	IncB/O/K/Z	*E. coli*	China	8 September 2023	An unused razor head	61%/95.34%	*bla*_NDM-13_, *bla*_SHV-12_, *aph (3′)-IIa*, *fosA*3, *rmt*B, *bla*_KPC-2_, *bla*_CTX-M-65_, *bla*_TEM-1B_	CP176052
pYZLc23-1_NDM-13_96k	IncB/O/K/Z	*E. coli*	China: Yangzhou	1 April 2019	Feces	61%/89.91%	*bla*_NDM13_, *aph (3′)-IIa*, *fosA*3	CP123269
pCSRM-NDM13	IncI1-I (Alpha)	*E. coli*	China: Hangzhou	2022	Wastewater	50%/95.97%	*bla*_NDM13_, *sul1*, *dfrA*12, *aph (3′)-Ia*, *aadA*2	CP123198
pNB4833-MCR	IncFIB (AP001918)/IncFII (pHN7A8)	*E. coli*	China: Ningbo	14 October 1984	Urine	18%/99.95%	*bla*_NDM13_, *sul1*, *dfrA*17, *aadA*5	CP118603
pHD12840-NDM13	IncX3	*E. coli*	China: Ningxia	27 September 2016	Pleural fluid	3%/99.85%	*bla*_NDM13_, *bla*_SHV-12_	CP143394
pZHDC33	IncX3	*E. coli*	NA	NA	NA	3%/99.85%	*bla*_NDM13_, *bla*_SHV-12_	KX094555

## Data Availability

The data can be obtained upon request from the corresponding author.

## Supplementary Materials

The following supporting information can be downloaded at https://www.mdpi.com/article/10.3390/antibiotics15040381/s1. Table S1: Information of 113 HC20-7952 strains from Enterobase analyzed in this study.
