# Supplementary material for: Genetic and Phenotypic Characterization of a Salmonella Enteritidis ST11 Clinical Isolate Carrying blaNDM-13 in Jiaxing City, China
Source: Antibiotics (Basel). 2026 Apr 9;15(4):381. doi: 10.3390/antibiotics15040381 (PMC13113174; doi:10.3390/antibiotics15040381)
Supplement: Supplementary file 1 [file antibiotics-15-00381-s001.zip › antibiotics-4192446-supplementary.pdf]

Table S1. Information of 113 HC20-7952 strains from Enterobase analyzed in this study.

Name	Source	Collection Date	Location	HC2	H20
54060	Human	2014-09-25	United Kingdom	24992	7952
FDA782494-B1	Environment	2012-12	Mexico	7952	7952
E2016019994	Human	2016-12	United States	59410	7952
PNUSAS010797	Human	2017-03	United States	69090	7952
PNUSAS016469	Human	2017-04	United States	76757	7952
PNUSAS017197		2017	United States	77218	7952
PNUSAS018381	Human	2017-06	United States	81912	7952
PNUSAS021939	Human	2017-05	United States	87410	7952
AUSMDU00006709	Human	2017	Australia	91278	7952
0472/SIS/2018-1T	Food	2018	Macao	221945	7952
192907	Human	2015-12	United Kingdom	152240	7952
SMG-18-2213		2018	United Kingdom	152690	7952
FD09A01	Livestock	2015-09-09	China	154303	7952
SE109	Human	2013-09	China	229062	7952
SE104	Human	2013-07	China	229063	7952
SJTUF12367v2	Human	2013-08	China	232248	7952
S14030	Human	2014	China	234680	7952
QLUY823	Poultry	2012	China	236372	7952
3.31-A5	Poultry	2018-03-31	China	181307	7952
3.31-B2	Poultry	2018-03-31	China	181306	7952
4.10-6C7	Poultry	2018-04-10	China	181337	7952
4.13-A8	Poultry	2018-04-13	China	181365	7952
1.05-1A6	Poultry	2018-01-05	China	181194	7952
1.13-3A1	Poultry	2018-01-13	China	181272	7952
3.6-1A3	Poultry	2018-03-06	China	181293	7952
3.31-C6	Poultry	2018-03-31	China	181308	7952
4.8-2A5	Poultry	2018-04-08	China	181336	7952
1.28-1A3	Poultry	2018-01-28	China	181278	7952
4.13-C1	Poultry	2018-04-13	China	181398	7952
3.11-A2	Poultry	2018-03-11	China	181299	7952
17ESS0233	Human	2017	China	239345	7952
Salmonella sp.	Human	2020-07	China	311094	7952

ZJSX2020-040					
Salmonella sp. ZJSX2020-039	Human	2020-07	China	311092	7952
18-9539-0116	Human	2018-07-25	Australia	328517	7952
SAL03541	Poultry	2017-02-08	China	330448	7952
SAL03530	Poultry	2017-02-08	China	330441	7952
SAL03483	Poultry	2018-05-21	China	330438	7952
SAL03574	Poultry	2018-05-21	China	330435	7952
SAL03571	Poultry	2017-02-08	China	330434	7952
SAL03551	Poultry	2017-02-08	China	330381	7952
SAL03509	Poultry	2013-01-12	China	330371	7952
SAL03503	Poultry	2018-05-21	China	330367	7952
SAL03493	Poultry	2018-05-21	China	330357	7952
W117	CJ	2023	China	350043	7952
GDUTNS_Y35	Poultry	2023-03-04	China	355716	7952
SAMEA112912687	Human	2013	Italy	361167	7952
NBFE-068	Human	2021	China	370927	7952
2338		2023	China	388833	7952
2197/DGP/2022-6T	Food	2022	Macao	389188	7952
GDUTNS_Y42	Poultry	2023-04-07	China	396506	7952
GDUTNS_Y47	Poultry	2023-05-02	China	396511	7952
GDUTNS_Y56	Poultry	2023-05-14	China	396530	7952
GDUTNS_Y103	Poultry	2023-10-27	China	396918	7952
GDUTNS_Y110	Poultry	2023-12-04	China	396931	7952
SE32	Human	2023-08-18	China	405310	7952
SE33	Human	2023-08-20	China	405325	7952
SE25	Human	2023-06-27	China	405328	7952
SE34	Human	2023-08-20	China	405333	7952
1448988	Human	2024-03	United Kingdom	405778	7952
SE49	Human	2020-07-13	China	405946	7952
SE1	Food	2021	China	416971	7952
SE19	Food	2023	China	417324	7952
SE20	Food	2023	China	417299	7952
19-9539-0301	Human	2019-05-22	Australia	449409	7952
1208_ZJQZ22_0088	Human	2022-05-20	China	461559	7952
SKLX126300	Human	2020	China	461571	7952
1201_ZJHZ21_0167	Human	2021-07-02	China	461580	7952
SKLX169990	Human	2021	China	461605	7952
SKLX169339	Human	2021	China	461618	7952
1208_ZJ22QZ0116SAL	Human	2022-08-08	China	461636	7952
1096_ZJNB22SAL003	Human	2022-01-01	China	461649	7952

SKLX162688	Human	2021	China	461682	7952
SKLX152891	Human	2021	China	461571	7952
SKLX147589	Human	2021	China	461770	7952
1204_ZJZS2020-022	Human	2020-05-24	China	461810	7952
SKLX145660	Human	2021	China	461866	7952
2023-0415-F	Human	2023	China	475749	7952
2017-0073-F	Human	2017	China	476109	7952
2023-0977-F	Human	2023	China	476706	7952
S02962_1092001743	Food	2020	Netherlands	482658	7952
TJ24WQ0205SAL	Poultry	2024	China	525045	7952
PNUSAS237601		2021-09	United States	557584	7952
1732384	Human	2025-04	United Kingdom	620429	7952
SMG-25-1118	Human	2025-05	United Kingdom	628429	7952
PNUSAS514422		2025	United States	629141	7952
1763983	Human	2025-06	United Kingdom	634001	7952
1765172	Food	2025-06	United Kingdom	634321	7952
2018AM-1702	ND	2018-01	United States	639882	7952
1791436	Food	2025-07	United Kingdom	628429	7952
AUSMDU00118867	Human	2025-06-05	Australia	646384	7952
AUSMDU00109205	Human	2024-10-03	Australia	646613	7952
PNUSAS531709	ND	2025	United States	652821	7952
PNUSAS537164		2025	United States	661601	7952
1829286	Food	2025-09	United Kingdom	666454	7952
GS20234288400190SM	Human	2023	China	668322	7952
GS093102202100024SM_S10_L001	Human	2021	China	668376	7952
GSJC2022006SM_S57	Poultry	2022	China	668387	7952
LANZHOU_GS093102-2024-00223	Human	2024	China	668443	7952
1847097	Human	2025-10	United Kingdom	634321	7952

GS093102202100072SM_S11_L001	Human	2021	China	673136	7952
PNUSAS554330		2025	United States	678845	7952
PNUSAS557641		2025	United States	681734	7952
1921221	Human	2026-02	United Kingdom	691109	7952
SAL31404	Human	2025-06-13	China	682080	7952
JX-2024-Oct-615	Human	2024-10-08	China	691794	7952
JX-2024-Oct-605	Human	2024-09-23	China	691785	7952
JX-2024-Sep-315	Human	2024-08-10	China	691790	7952
JX-July-101	Human	2024-05-25	China	691791	7952
JX-2024-Oct-413	Human	2024-09-06	China	691796	7952
124209	Human	2025-07-13	China	691787	7952
368350SM	Human	2025-09-13	China	691803	7952
32007SM	Human	2025-01-16	China	691795	7952
25JX-109	Human	2025-05-12	China	691802	7952
